# Combined Deltoid and Spring Ligament Reconstruction Using the Quadrangular Construct: Our Experience and Review of the Literature

**DOI:** 10.7759/cureus.50361

**Published:** 2023-12-11

**Authors:** Pradeep Moonot, Shubham Dakhode, Nikhil Karwande, Prashant Pawar

**Affiliations:** 1 Orthopaedics, Mumbai Knee Foot Ankle Clinic, Mumbai, IND; 2 Orthopaedics, Sir H. N. Reliance Foundation Hospital, Mumbai, IND; 3 Orthopaedics, Breach Candy Hospital, Mumbai, IND

**Keywords:** chronic ankle instability, ankle arthroscopy, internal brace, ankle reconstruction, chronic deltoid injury, quadrangular construct, medial ankle instability, spring ligament, deltoid ligament

## Abstract

Background

A combined reconstruction of chronic deltoid and spring ligament insufficiency is uncommon. Our study aims to share our experience in treating post-traumatic, chronic deltoid, and spring ligament insufficiency using the "quadrangular construct" technique.

Material and methods

Five patients who had post-traumatic combined deltoid and spring ligament insufficiency were included in the study. All patients reported a "giving-way" sensation. Preoperatively, each patient underwent weight-bearing radiographs of the ankle and foot. The talo-first metatarsal angle and hindfoot alignment angle were noted. The superficial deltoid ligament was repaired using a suture anchor augmented with Internal Brace^TM^ (Arthrex, Naples, USA) FiberTape® to form a quadrangular construct that anatomically mimics various components of the deltoid-spring ligament complex. Due to the associated excessive heel valgus, three patients also underwent medial displacement calcaneum osteotomy. Additionally, one patient required lateral ligament repair, and another patient required syndesmotic stabilization. The American Orthopaedic Foot and Ankle Society (AOFAS) hindfoot score was used to evaluate preoperative and postoperative ankle function.

Results

All five patients were followed up for a mean of 20 months (range: 12-24 months). The mean preoperative talo-first metatarsal angle improved from 8.46 degrees to 4.84 degrees. The preoperative mean hindfoot alignment angle was reduced from 10.9 to 5.76 degrees postoperatively. One patient had irritation due to the anchor, which needed removal after one year. Postoperatively, no patients re-experienced the feeling of "giving way". The AOFAS scores postoperatively showed two patients as excellent, two as good, and one as fair. All the patients returned to their pre-injury work.

Conclusion

We have developed a technique for combined deltoid and spring ligament reconstruction using a quadrangular construct. This technique helps to restore anatomical stability, is safe, easily reproducible, and has shown positive short-term results in follow-up. The level of evidence is one of the methods used to categorize the quality and reliability of research, and our study falls under the category of level IV evidence.

## Introduction

The deltoid ligament is the strongest ligament on the medial side of the ankle, spanning between the talocrural and the talocalcaneonavicular joints. It is closely related to the tibialis posterior tendon, sustentaculum tali, and calcaneonavicular (spring) ligament; together, they form a crucial complex on the medial aspect that stabilizes the ankle and subtalar joints. [1]. While most ankle sprains involve damage to the lateral ankle ligament complex, injuries to the medial ankle ligaments are more common than generally believed [2, 3]. Unfortunately, deltoid injuries are often neglected, overlooked, and undertreated, leading to chronic insufficiency and additional strain on other tissues, particularly the spring ligament. This can result in combined deltoid and spring ligament insufficiency, for which there are few treatment methods mentioned in the literature. In this study, we discuss our experience in treating post-traumatic, chronic deltoid, and spring ligament insufficiency using a technique called the "quadrangular construct" (combined deltoid and spring ligament reconstruction), which utilizes FiberTape® and suture anchor fixation to anatomically resemble the ligament complex.

This article was previously presented as a poster at the 2023 Annual Indian Foot & Ankle Society Conference (IFASCON) on August 27, 2023, and an abstract was also selected for presentation at the 2023 Société Internationale de Chirurgie Orthopédique et de Traumatologie (SICOT) Conference on November 22, 2023.

## Materials and methods

This is a prospective study that includes five patients with chronic deltoid injuries who underwent surgical management using the quadrangular construct technique (combined deltoid and spring ligament reconstruction) with Internal Brace™ (Arthrex Inc., Naples, FL) FiberTape® and suture anchor fixation. All patients had a history of trauma or a sprain. Prior to being referred to us, two patients were managed conservatively, and the deltoid ligament was not addressed during the initial surgery for the remaining three patients. All patients presented with medial ankle pain, swelling, and a sensation of instability while walking. Physical examination during weight bearing revealed tenderness at the site of attachment of the deltoid over the medial malleolus, a collapsed medial arch of the foot, and hindfoot valgus. The hindfoot valgus was correctable with varus stress. Weight-bearing radiographs of the ankle in the anteroposterior (AP), lateral, and Saltzman views were performed for all patients. Deltoid insufficiency was indicated by valgus talar tilt on standing anteroposterior radiographs of the ankle. The Saltzman view was used to quantify hindfoot valgus, with a deformity diagnosed if the angle was greater than 5 degrees [4]. Flat foot deformity was quantified using the talo-first metatarsal angle on lateral weight-bearing radiographs, with a diagnosis made if the angle was greater than four degrees [5]. An MRI was performed for all patients and revealed deltoid and spring ligament injuries. Informed consent was obtained from all patients, and the study was approved by the Institutional Ethics Committee of Sir H.N. Reliance Foundation Hospital & Research Centre, Mumbai, India, with the approval number HNH/IEC/CR/ORTH/19.

Anatomy

**Figure 1:**
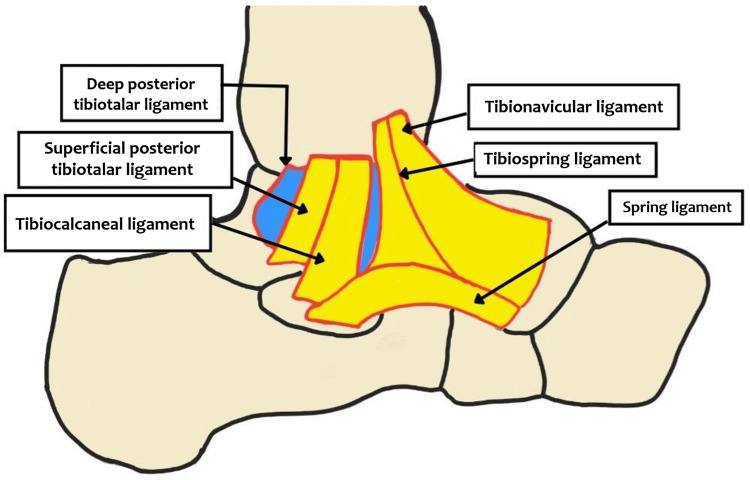
The superficial layer of the deltoid ligament This is the authors' diagrammatic representation of different components of the superficial layer of the deltoid ligament and the broad insertion of the superficial deltoid over the spring ligament

In order to successfully perform an anatomical repair or reconstruction, it is essential to have a thorough understanding of the deltoid ligament complex. This multifascicular ligament plays a crucial role in stabilizing the medial side of the ankle and preventing talar abduction and translation at the talocrural joint. It extends from the medial malleolus to the naviculum, talus, and calcaneum [6]. The deltoid ligament complex consists of a deep layer and a superficial layer, which are divided by a layer of fat between them. There are four components of the superficial layer, which extend from the tibia to the navicle (the tibionavicular component), the tibia to the spring ligament (the tibiospring component), the tibia to the sustentaculum tali (the tibiocalcaneal component), and the tibia to the talus (the superficial tibiotalar component) (Figure 1).

**Figure 2:**
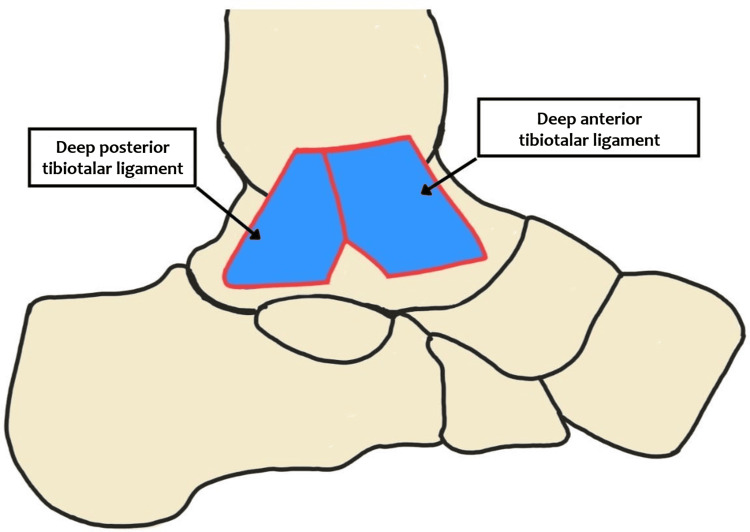
Different components of the deep layer of the deltoid ligament This is the authors' diagrammatic representation of the two components of the deep layer of the deltoid ligament.

The deep layer has two components that extend from the tibia to the talus, viz., the deep posterior and deep anterior tibiotalar components [7] (Figure 2).

In particular, the deep deltoid prevents external rotation of the talus, and the superficial deltoid prevents the hindfoot from eversion [8]. The spring ligament, or calcaneonavicular complex, which connects the navicular bone with the sustentaculum tali, is made up of three ligaments which are inferoplantar longitudinal, medioplantar oblique, and superomedial ligaments [9]. Since the superficial deltoid receives a wide insertion into the spring ligament, it is possible that both ligaments work together to maintain the medial ankle joint, and the deltoid and spring ligament may not be functionally distinct from one another [10] (Figure 1). Hence, the spring ligament may also wear out over time in cases of chronic deltoid injury; for this reason, the spring ligament must be addressed during surgical repair of chronic deltoid injuries.

Surgical technique 

**Figure 3:**
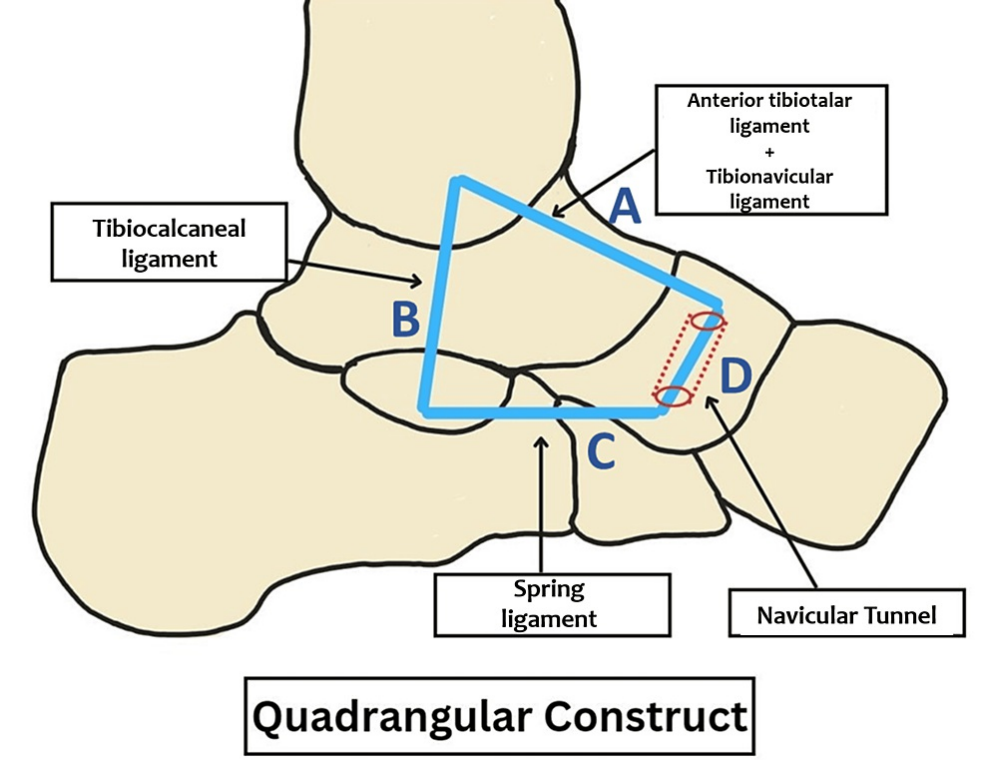
Quadrangular construct This is the authors' diagrammatic representation of the quadrangular construct. Side A forms the anterior tibiotalar and tibionavicular parts of the deltoid complex. Side B forms the tibiocalcaneal component. Side C forms the spring ligament, extending from the plantar side of the naviculum to the sustentaculum tali. Side D represents the navicular tunnel.

The patients received spinal anesthesia and were placed in a supine position. After administering antibiotic prophylaxis, a tourniquet was placed on the thigh and elevated. Ankle arthroscopy was performed to confirm the medial ankle instability. The intra-articular lesions, hypertrophied synovium, and fibrous tissue were debrided. A curved incision was made 1 cm proximal to the tip of the medial malleolus, extending to the medial aspect of the navicular bone. The tributaries of the saphenous vein and saphenous nerve were isolated to reach and expose the superficial fascia. The superficial layer of the deltoid was revealed after exposure and retraction of the tibialis posterior tendon. Differentiating between the two components (superficial and deep) of the deltoid can be difficult because of their close structural relationship. The superficial deltoid ligament was transversely incised approximately 5 mm from the medial malleolus. The proximal severed end of the superficial deltoid ligament and the periosteum of the medial malleolus were sharply dissected and retracted, exposing the bony medial malleolus. The superficial deltoid was repaired using a 5.5 mm metallic anchor inserted into the medial malleolus. The navicular tuberosity and sustentaculum tali were then exposed. The mid-portion of the FiberTape® with a 3.5 mm SwiveLock (Arthrex Inc.) was inserted into the sustentaculum tali, leaving behind two ends of FiberTape®. A bony tunnel of size 3.5 mm was made in the navicular bone, and one end of the FiberTape® was threaded through it in the plantarodorsal direction. The medial malleolus was drilled with a 3.5 mm cannulated drill for the insertion of a 4.5 mm SwiveLock (Arthrex Inc.). After keeping the ankle in a neutral position, the ends of both arms were now passed through the 4.5 mm SwiveLock eyelet and then anchored into the medial malleolus. Adequate tension was kept in the FiberTape® to form a “quadrangular construct” [11] (Figure 3).

The proximal severed end of the superficial deltoid ligament and the periosteum of the medial malleolus were sutured over the distal end with the FiberWire® (Arthrex Inc.) of the anchor, which was already placed at the start. The postoperative leg was immobilized in a neutral ankle position using a short-leg plaster slab.

Postoperative protocol

Patients were initially protected with a below-knee posterior slab for two weeks until suture removal. Following suture removal, a below-knee plaster cast was applied for an additional two weeks. After the plaster cast was removed, ankle range of motion exercises were initiated, with a focus on avoiding eversion. Gradual partial weight bearing was introduced after six to eight weeks using a removable walker boot. Patients were able to walk with full weight bearing within three months.

Evaluation

All patients were regularly followed up, and at the last follow-up, weight-bearing ankle radiography and the Saltzman view [4] were performed to compare with preoperative radiographs. The American Orthopedic Foot and Ankle Society's (AOFAS) [12] scoring system for the ankle-hindfoot was used to evaluate preoperative and postoperative ankle performance.

## Results

All five patients were followed up for a mean of 20 months (range: 12-24 months). All five patients underwent the quadrangular construct. Medial displacement calcaneal osteotomy (MDCO) was performed in three patients due to associated excessive heel valgus. As an additional procedure, lateral ligament repair was needed in one patient, and one patient required syndesmotic stabilization (Table 1).

**Table 1:** Patient demographics F: female; M: male; MDCO: medial displacement calcaneal osteotomy

S. No.	Age/Sex	Time since injury	Procedure performed
1.	39/F	6 months	Quadrangular construct + MDCO
2.	50/F	8 months	Quadrangular construct + MDCO
3.	45/M	3 months	Quadrangular construct + MDCO + Syndesmotic fixation
4.	28/M	5 months	Quadrangular construct + Lateral ligament repair
5.	32/F	4 months	Quadrangular construct

The mean talo-first metatarsal angle on preoperative weight-bearing radiographs was 8.46 degrees for the injured side and 4.98 degrees for the healthy side. The preoperative mean hindfoot alignment angle in the Saltzman view was 10.9 degrees for the injured side and 5.46 degrees for the healthy side [4]. Postoperatively, for the injured side, the mean talo-first metatarsal angle and the mean hindfoot alignment angle were 4.84 degrees and 5.76 degrees, respectively. A paired t-test was applied to determine the statistical significance of the observed changes. For the talo-first metatarsal angle, it was found to be statistically significant with a p-value of <0.05 (0.03). For hindfoot valgus, the p-value was not statistically significant (p~0.05). This was because only three patients underwent MDCO due to significant hindfoot valgus and experienced a greater change in hindfoot valgus postoperatively, while the remaining two patients did not have significant valgus (Table 2).

**Table 2:** Comparison of preoperative and postoperative radiologic alignment in degrees The change in the talo-first metatarsal angle was statistically significant in all patients (p<0.05). Since MDCO was done in only three patients, changes in hindfoot valgus were overall not statistically significant (p~0.05). *MDCO is not done. MDCO: medial displacement calcaneal osteotomy

Sr. No		1	2	3	4	5	p-value
Talo-first metatarsal angle	Preoperative	10.1	9.4	11.3	6.4	5.1	<0.05 (0.03)
	Postoperative	5.4	4.2	4.8	5.6	4.2	
Hindfoot valgus	Preoperative	12.8	13.3	14.6	7.5*	6.3*	~0.05(0.051)
	Postoperative	4.8	5.2	6.1	6.9*	5.8*	

All patients experienced relief from pain post surgery. Two patients experienced some swelling around their ankles in the initial months, but it gradually resolved. One patient experienced irritability at the anchor insertion site, which needed to be removed one year after surgery. No other complications were noted. Postoperatively, none of the patients re-experienced the feeling of "giving way", which was a major concern for the patients. Ankle and hindfoot performance was evaluated using AOFAS scores. Preoperatively, all patients had a poor (<69) AOFAS score, whereas postoperatively, at the latest follow-up, two patients had an excellent outcome (90-100), two had a good outcome (80-89), and one had a fair outcome (70-79) [12]. All the patients could perform their activities of daily living and recreational activities independently and return to their pre-injury work (Table 3).

**Table 3:** Postoperative parameters Postoperatively, no patients experienced pain or the feeling of "giving way." (+) present; (-) absent * Two patients had mild swelling around the medial malleolus in the initial months, but it gradually resolved. # Irritability due to a metal anchor that was removed after one year post surgery. ** The AOFAS score is graded as poor (<69), fair (70–79), good (80–89), and excellent (90–100) [12]. AOFAS: American Orthopedic Foot and Ankle Society

S. No.	Pain		Postoperative complications	Feeling of "giving way"		AOFAS score**		Return to work
	Preoperative	Postoperative		Preoperative	Postoperative	Preoperative	Postoperative	
1.	(+)	(-)	Nil	(+)	(-)	25	85	(+)
2.	(+)	(-)	Swelling around the ankle *	(+)	(-)	34	81	(+)
3.	(+)	(-)	Swelling around the ankle *	(+)	(-)	21	79	(+)
4.	(+)	(-)	Irritability due to metal anchor ^#^	(+)	(-)	58	98	(+)
5.	(+)	(-)	Nil	(+)	(-)	54	95	(+)

## Discussion

**Figure 4:**
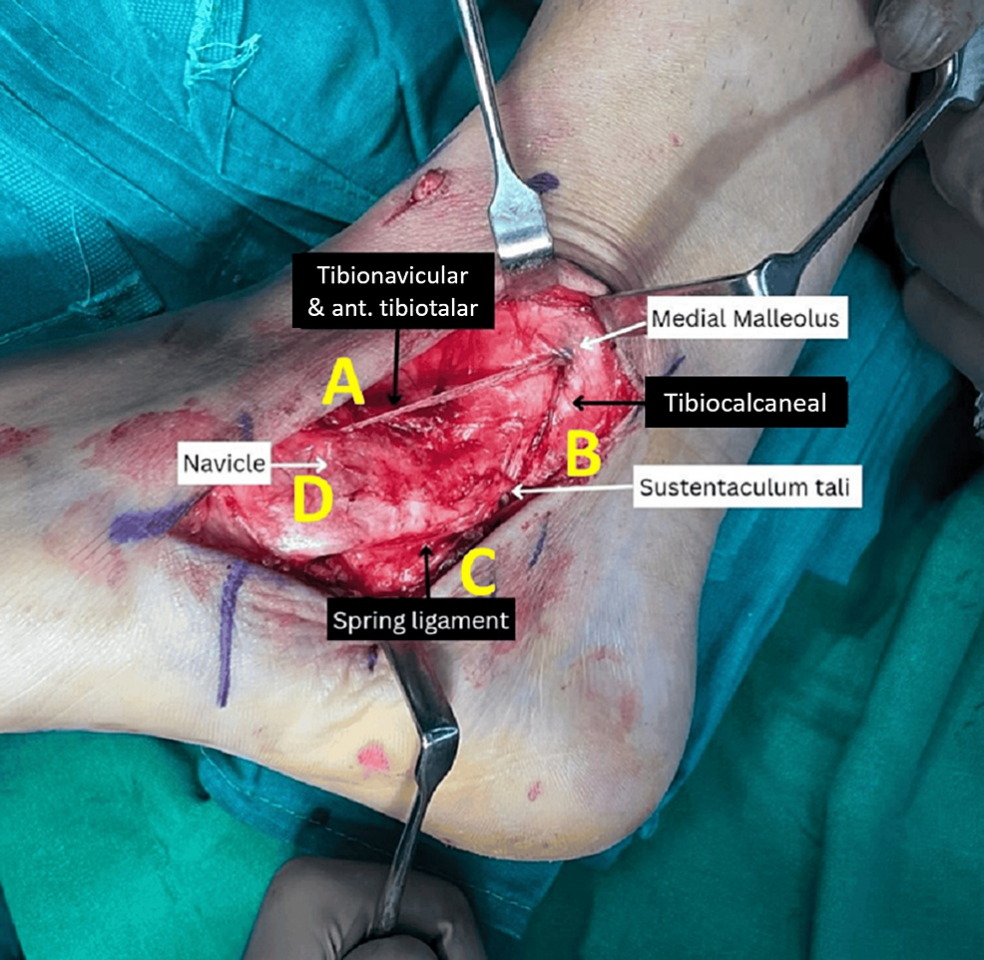
Intraoperative image showing various components of the quadrangular construct ant: anterior

The deltoid ligament is primarily responsible for stabilizing the ankle joint, whereas the spring ligament is mostly responsible for supporting the medial longitudinal arch. Due to the deltoid's wide insertion over the spring ligament, Hintermann et al. [10], Kelikian [13], Milner and Soames [14], and others found that the superficial layer of the deltoid ligament is closely related to the spring ligament. Both ligaments are interwoven to provide medial stability and support for the talar head, and they may not be functionally distinct from one another. Hence, it is clear that both ligaments must be treated at the same time. Studies that discuss management strategies for combined deltoid and spring injuries are uncommon. We outline a practical approach to treating both deltoid and spring ligament dysfunction using our quadrangular construct technique [11] (Figure 3). In our series, we have used suture anchors to repair the superficial deltoid ligament. Following this, we augmented this repair with FiberTape® (Internal BraceTM, Arthrex Inc.) [15], utilizing the quadrangular construct [11]. Side A of the construct, from the medial malleolus to the dorsal side of the naviculum, forms the anterior tibiotalar and tibionavicular parts of the deltoid complex. Side B, which extends from the medial malleolus to the sustentaculum tali, forms the tibiocalcaneal component. Side C represents the spring ligament extending from the plantar side of the naviculum to the sustentaculum tali (Figure 3). As a result, each side of the quadrangle anatomically mimics different parts of the deltoid-spring ligament complex, providing good stability (Figure 4).

**Figure 5:**
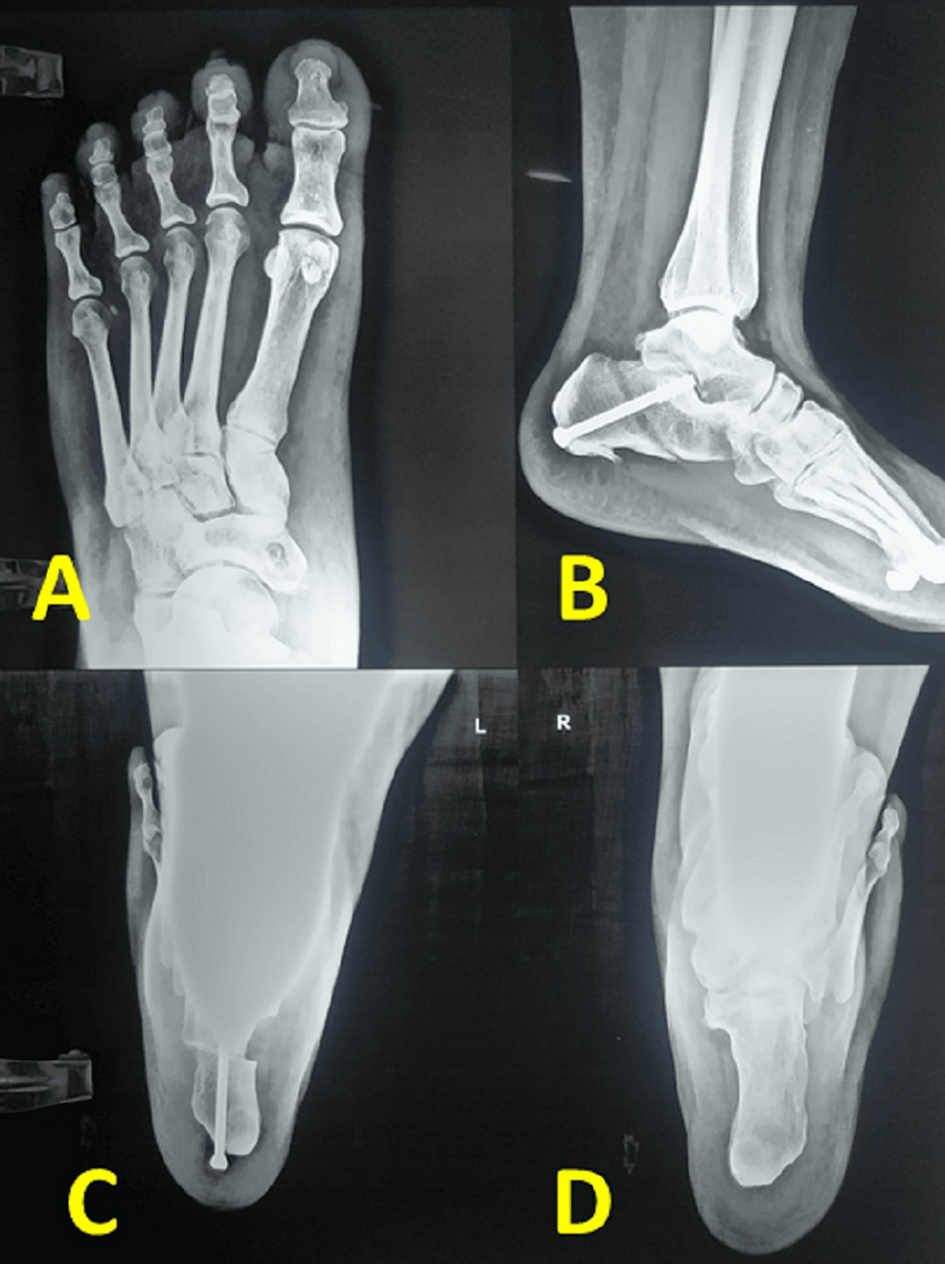
Postoperative X-ray of a patient who had undergone the quadrangular construct and medial displacement calcaneum osteotomy Figure A shows a hole around the navicular tuberosity, indicating the navicular tunnel of a quadrangular construct. Figures B and C show a medial displacement calcaneum osteotomy fixed with a single screw. Figure D shows a comparative Saltzman view of the normal side [4].

**Figure 6:**
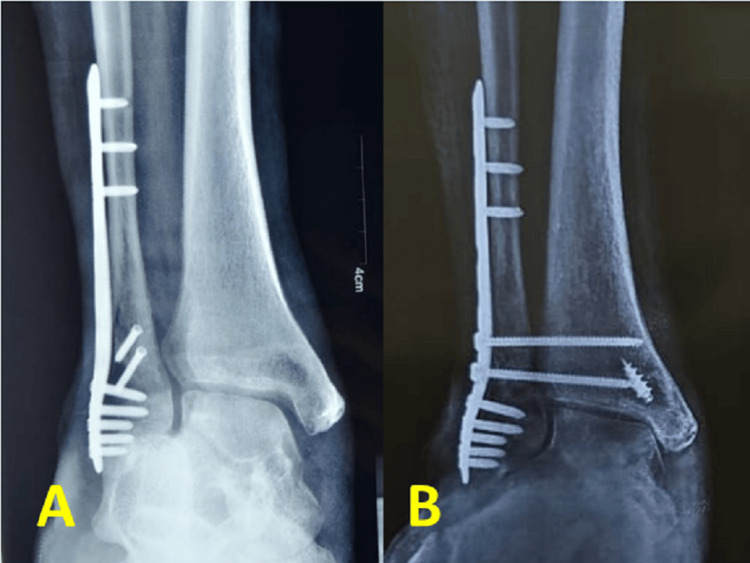
Preoperative (A) and postoperative X-rays (B) of a patient who underwent the quadrangular construct with syndesmotic stabilization and medial displacement calcaneum osteotomy.

Pellegrini et al. [15] used Internal Brace^TM^ augmentation in 13 patients and found good functional outcomes for chronic deltoid injury repair. Only the tibiocalcaneal and tibiotalar components of the deltoid-spring complex were comparable in their construct. However, our design differs from theirs as it includes three distinct components in addition to spring ligament augmentation. Nery et al. [16] used two FiberTape® to describe a novel technique for combined deltoid and spring ligament reconstruction in patients with adult-acquired flatfoot. They had 10 patients, all of whom experienced pain relief and returned to their previous activities with an improvement in their AOFAS score. Two components were reconstructed in their technique. One was the anterior tibiotalar and the other was the tibiocalcaneal, in addition to the spring ligament. Their patients also had posterior tibial tendon dysfunction as a result of the associated adult-acquired flatfoot, necessitating flexor digitorum longus (FDL) transfer. Grunfeld et al. used a peroneus longus allograft with FDL transfer to reconstruct the combined deltoid-spring ligament in three patients [17]. There are several techniques described in the literature that describe isolated deltoid or spring ligament reconstruction with variable results. Wiltberger and Mallory were the first to describe a posterior tibial tendon graft for deltoid ligament reconstruction in 1972 [18]. However, due to the risk of posterior tendon insufficiency, it cannot be used as a graft. In an effort to emulate the mechanism of the anterior tibiotalar ligament, Bohay et al. employed the autologous flexor hallucis longus tendon and fastened it to the medial side of the talus in cases of chronic deltoid insufficiency, which had a fair outcome [19]. In severe cases of adult-acquired flatfoot, Ellis et al. used an autologous peroneus longus graft for deltoid repair in 2010 [20]. Myerson et al. employed hamstring allografts to reconstruct the tibiotalar and tibiaocalcaneal components of the deltoid ligament in five patients with severe flatfoot deformity, with a minimum follow-up of 20 months following surgery. The functional scores of all five patients were on par with age-appropriate normative ratings [21]. Hintermann rebuilt the tibionavicular ligament using an autologous plantaris tendon and placed it between the naviculum and the medial malleolus. [22]. Robinson et al. employed an autologous posterior tibial tendon for the augmentation of the spring ligament [23]. For spring ligament reconstruction in patients with adult-acquired flatfoot, Ellis et al. used an Achilles allograft in 2010 [24]. Both autografts and allografts are used in all of the techniques mentioned for reconstruction. Unfortunately, these have several disadvantages, including donor site morbidity, difficulty harvesting grafts for autografts, and poor availability and cost restrictions for allografts. In these situations, using our method of reconstruction aids in addressing these issues as it is safe and easily reproducible. In our series, three patients with increased heel valgus had undergone MDCO (Figure 5), one patient underwent syndesmotic stabilization (Figure 6), and one patient underwent lateral ligament repair.

It is challenging to distinguish between the superficial and deep deltoid ligaments and repair them independently. Reattaching the superficial deltoid to its original location is a crucial step in our procedure because it allows the native deep deltoid ligament to recover properly. The construct is made superficial to the reattachment of the superficial deltoid ligament. This makes the construct extra-articular, eliminates the risk of joint irritation, and also acts as a check-rein to allow the reattached native ligament to heal. Although the quadrangular construct cannot completely replace native ligaments, it can serve as an adjunct to repair since it anatomically resembles several components of the deltoid ligament and also adds support to the spring ligament. As a result, we think adding this construct is safe, and it will assist in providing the necessary stability on the medial aspect of the ankle in cases of chronic deltoid and spring ligament insufficiency.

Injuries to the deltoid and spring ligaments are often part of a complex injury pattern, yet they are frequently overlooked or not given sufficient attention during the treatment process. Despite the fact that the senior author of the study has a dedicated foot and ankle practice at prominent tertiary healthcare centers, the number of patients who present or are referred for such injuries at a stage where the deformity is correctable and joint preservation through ligament reconstruction and supplementary procedures is feasible remains quite limited. Consequently, it proves challenging to gather a substantial sample size. Also, as these injuries are part of a complex injury pattern, they do not occur in isolation but are associated with other injuries or deformities. Therefore, for complete correction of deformity, supplementary procedures are often necessary, which are also done in our patients. Nevertheless, it's important to acknowledge that the cohort size is small, which is a limitation of the study. However, the study's primary objective is to offer valuable preliminary data that can serve as a foundation for generating hypotheses in future studies with a more extensive sample size, possibly conducted across multiple healthcare centers. Also, a brief follow-up and the lack of a control group for comparison are some other limitations of the study.

## Conclusions

It is uncommon and challenging to treat post-traumatic chronic deltoid and spring ligament insufficiency. For adequate stability and support of the medial longitudinal arch, we advise that the reconstructive procedure should ensure anatomical restoration of the medial ligament complex. Our technique of combined deltoid and spring ligament reconstruction using the quadrangular construct aids in the anatomical restoration of stability. This technique is easily reproducible and safe and has demonstrated positive outcomes in short-term follow-ups.
